# Edoxaban Resolved a Left Atrial Thrombus Intruding From the Right Lower Pulmonary Vein and Increased Cerebral Blood Flow: A Case Report

**DOI:** 10.7759/cureus.111815

**Published:** 2026-06-30

**Authors:** Hidekazu Takeuchi

**Affiliations:** 1 Internal Medicine (Cardiology), Takeuchi Naika Clinic, Ogachi-Gun, JPN

**Keywords:** brain perfusion spect, cerebral blood flow, dementia, edoxaban, pulmonary vein thrombosis

## Abstract

Pulmonary vein thrombosis (PVT) is thought to be rare; however, since 2012, the author has used 64- or 80-slice multidetector computed tomography (64- or 80-MDCT) to describe several cases of PVT. Some reports have involved the use of transesophageal echocardiography (TEE) to characterize PVT. Notably, the mechanism of how edoxaban affects thrombi in the pulmonary vein and left atrium and cerebral blood flow is poorly understood.

An 85-year-old female with hypertension had a thrombus in her right lower pulmonary vein (RLPV) that intruded into the left atrium and exhibited snake-like characteristics when examined using TEE. The left atrium portion of the thrombus could be accurately imaged via TEE but not via 80-MDCT because it was thin and moved periodically. After six months of edoxaban treatment, the thrombus in the left atrium disappeared, as demonstrated by TEE, and cerebral blood flow increased, as measured by brain perfusion single-photon emission computed tomography (SPECT). Blood flow and a moving thrombus in the heart can be evaluated using TEE. Assessments via 80-MDCT alone may underestimate the incidence of a thrombus. Edoxaban ameliorated cerebral blood flow after six months of treatment, as measured via brain perfusion SPECT. Of note, treatment might reduce morbidity and prevent complications.

## Introduction

Ischemic stroke is a crucial clinical problem. Atrial fibrillation (AF) is the most common type of arrhythmia found in clinical practice, and it is encountered at elevated frequencies in elderly individuals. Non-valvular AF affects approximately two million Americans and is an independent risk factor for ischemic stroke [1].

Significant complications associated with AF include ischemic stroke and mortality. Although AF is related to a five-fold increase in the risk of ischemic stroke [2] and a two-fold increase in the risk of mortality [3], the incidence of ischemic stroke and mortality has been shown to decrease by two-thirds in response to direct oral anticoagulants (DOACs) [4]. AF is associated with approximately 15-20% of cases of ischemic stroke in individuals who do not receive treatment, and the annual risk of stroke is as high as 5% in patients with non-valvular AF who do not receive treatment. Recently, the timing of DOAC initiation after acute ischemic stroke for patients with AF was thoroughly investigated [5].

The left atrial appendage is a primary reservoir for the formation of atrial thrombi [6].

Pulmonary vein thrombosis (PVT) is thought to be rare, and the main causes of PVT are thought to be surgery of the lung, such as lung lobectomy [6] or bilobectomy, radiofrequency catheter ablation, and certain lung tumors [7].

However, since 2012, the author has published several case reports in which 64-slice multidetector computed tomography (64-MDCT) has been used to describe PVT in elderly patients without a history of lung cancer or thoracic surgery [8,9], suggesting that PVT is not rare. Moreover, in 2014, the author reported that PVT and left atrial thrombi are common in elderly individuals [10]. PVT has the potential to cause systemic embolization, leading to conditions such as ischemic stroke [11] and acute myocardial infarction [12].

Cerebral blood flow is reduced in patients with Alzheimer’s disease and can be estimated using brain perfusion single-photon emission computed tomography (SPECT) [13]. In Japan, brain perfusion SPECT results have been analysed using the easy Z score Imaging System (eZIS) [14].

Edoxaban is an oral, reversible, direct factor Xa inhibitor with a rapid onset of action; it is administered once daily and has proven antithrombotic efficacy. The author reported that six months of treatment with DOACs improved cerebral blood flow, as estimated using brain perfusion SPECT [15]. There have been several reports in which thrombi in the pulmonary vein have been described using transesophageal echocardiography (TEE) [16,17].

This case report describes a left atrial thrombus that intruded from the right lower pulmonary vein (RLPV) and had a root in a branch of this vein. Edoxaban resolved the thrombus and increased cerebral blood flow without the occurrence of ischemic stroke.

## Case presentation

The patient was an 85-year-old, normally developed and nourished Japanese female with hypertension, in no apparent distress, and walking without a cane. The patient had no memory distress and was calm. The patient could eat meals by herself, and her son accompanied her to the hospital every month. To check for PVTs, the patient underwent cardiac CT and TEE, and the patient was treated with edoxaban to treat PVTs; then, SPECT was performed to assess changes in cerebral blood flow. Heart sounds were clear, and her heart rhythm was regular without murmurs. Breath sounds were normal with no crackles or wheezes. The patient’s BMI was 23. A chest roentgenogram did not confirm the presence of lung cancer or cardiomegaly. The patient had not been treated with DOACs or warfarin. ECG indicated normal sinus rhythm, a normal axis, and flat T waves in II, III, and aVF; the patient’s heart rate was 79 beats/min. Her serum D-dimer concentration was 1.0 μg/ml (normal: < 1.0 μg/ml), and her protein S activity was 79% (normal: 60-127%). The patient’s protein C activity was 124% (normal: 64-135%), and her homocysteine and BNP levels were 10.5 nmol/mL (normal: 5-15 nmol/mL) and 52 pg/ml (normal: 0.0-18.4 pg/ml), respectively. With respect to brain perfusion SPECT results, the calculated eZIS values were 0.67 for severity (normal: < 1.19), 0.8% for extent (normal: < 14.2%), and 0.04 for ratio (normal: < 2.22).

To evaluate the thrombus in the left atrium and pulmonary vein, TEE was performed; this examination revealed a snake-like thrombus in the left atrium (Figure 1A) that appeared to connect to a thrombus in the RLPV. To verify the presence of a thrombus in the RLPV, 80-slice multidetector computed tomography (80-MDCT) was conducted. This procedure confirmed the presence of a thrombus in the RLPV but could not clearly illustrate the thrombus in the left atrium (Figures 1B, 1C). 

**Figure 1:**
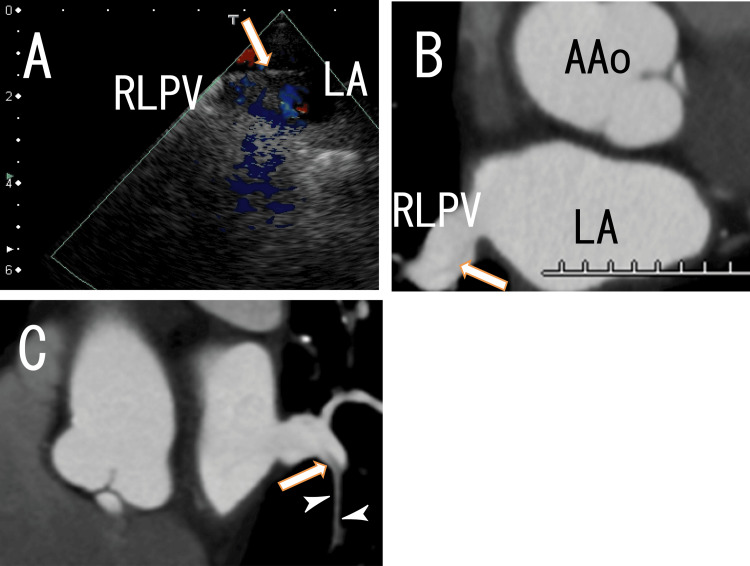
Transesophageal echocardiography (TEE) and 80-slice multidetector computed tomography (80-MDCT) images depicting a thrombus TEE images depicting a thrombus moving like a snake in the left atrium that appeared to be connected to a thrombus in the right lower pulmonary vein (RLPV) (arrow) (A). Axial images from an 80-MDCT scan that revealed a thin, obscure, dark thrombus in the RLPV (arrow). No thrombus was detected in the left atrium (B). Sagittal images from an 80-MDCT scan revealed a thrombus in the RLPV (arrow); these images depict a dark branch of the RLPV, indicating complete occlusion of this branch by the thrombus (arrow heads) (C). AAo; ascending aorta, LA; left atrium, RLPV; right lower pulmonary vein

Furthermore, video images obtained via TEE revealed a snake-like thrombus in the left atrium (Video 1) that appeared to be rooted in the RLPV and moved periodically with heartbeats.

**Video 1:** Transesophageal echocardiography (TEE) images of a snake-like thrombus in the left atrium TEE images revealed a snake-like thrombus in the left atrium. The thrombus in the right lower pulmonary vein (RLPV) did not move and may have been attached to the vessel wall; however, the thrombus in the left atrium moved like a wave.

Blood flow through the right upper pulmonary vein was observed as a red area (Video 2); blood flowed around the snake-like thrombus.

**Video 2:** Transesophageal echocardiography (TEE) images of blood flow from the right upper pulmonary vein TEE images of blood flow from the right upper pulmonary vein, which reached the entrance of the RLPV and surrounded the snake-like thrombus.

Edoxaban (30 mg, once daily) treatment was initiated. After three months of edoxaban treatment, the thrombus in the left atrium had narrowed and shortened (Figure 2A and Video 3). Brain perfusion SPECT was performed, and the calculated eZIS values, which were 0.54 for severity, 0.13% for extent, and 0.01 for ratio, indicated increased cerebral blood flow.

**Figure 2:**
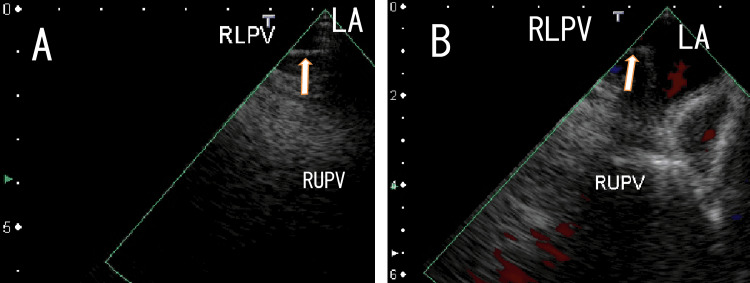
Transesophageal echocardiography (TEE) images obtained after three and six months of edoxaban treatment TEE images obtained after three months of edoxaban treatment revealed a narrowed and shortened thrombus in the left atrium that appeared to be connected to a thrombus in the RLPV (arrow) (A). TEE images obtained after six months of edoxaban treatment revealed no thrombus in the left atrium; however, the connected thrombus in the RLPV remained present (arrow). The red area in the left atrium indicated blood flow from the right upper pulmonary vein (B). LA; left atrium, RLPV; right lower pulmonary vein, RUPV; right upper pulmonary vein.

**Video 3:** Transesophageal echocardiography (TEE) images obtained after three months of edoxaban treatment TEE images obtained after three months of edoxaban treatment revealed a narrowed and shortened thrombus in the left atrium, which exhibited no wave-like motion and appeared to be connected to a thrombus in the right lower pulmonary vein (RLPV).

After six months of edoxaban treatment, the thrombus in the left atrium had disappeared (Figure 2B and Video 4).

**Video 4:** Transesophageal echocardiography (TEE) images after six months of edoxaban treatment TEE images after six months of edoxaban treatment revealed no thrombus in the left atrium; however, the connected thrombus in the right lower pulmonary vein (RLPV), which did not exhibit wave-like motion, was present. Blood flow was detected on both sides of the thrombus in the RLPV.

Edoxaban did not resolve the thrombus in the RLPV, as assessed using 80-MDCT (Figures 3A, 3B).

**Figure 3:**
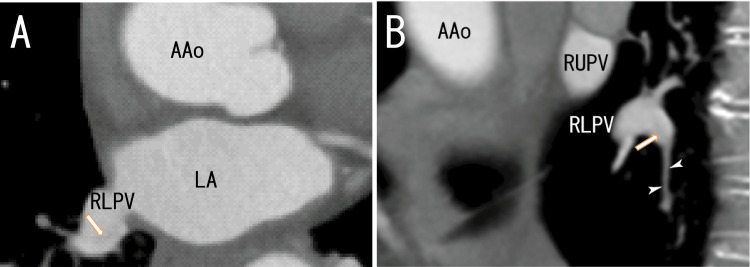
Images from an 80-MDCT scan after six months of edoxaban treatment Axial images from an 80-MDCT scan after six months of edoxaban treatment revealed a thrombus in the right lower pulmonary vein (RLPV) (arrow) that did not appear to significantly change after treatment (A). Sagittal images from an 80-MDCT scan after six months of edoxaban treatment revealed a thrombus in the RLPV (arrow), which did not appear to significantly change after treatment (arrow) (B). 80-MDCT; 80-slice multidetector computed tomography, AAo; ascending aorta, LA; left atrium, RLPV; right lower pulmonary vein, RUPV; right upper pulmonary vein

Brain perfusion SPECT was performed again, and the calculated eZIS values, which were 0.39 for severity, 0.0% for extent, and 0.00 for ratio, revealed a further increase in cerebral blood flow.

The initial laboratory results are summarized in Table 1, and the main changes induced by treatment are summarized in Table 2.

**Table 1:** Initial laboratory results Hb: Hemoglobin; Plt: Platelet; BUN: Blood urea nitrogen; TG: Triglyceride; HDL-C: High-density-lipoprotein cholesterol; LDL-C: Low-density-lipoprotein cholesterol; CRP: C-reactive protein; FT4: Free thyroxine (3,5,3′,5′-tetraiodo-L-thyronine); FT3: Free 3,3′,5′-triiodo-L-thyronine; TSH: Thyroxine-stimulating hormone; ApoA1: Apolipoprotein A1; ApoA2: Apolipoprotein A2; ApoB: Apolipoprotein B; ApoC2: Apolipoprotein C2; ApoC3: Apolipoprotein C3; ApoE: Apolipoprotein E

Item	Normal range	Results
Hb	14~18 g/dL	13.1
Plt	15~40×10^4/μL	24.3
Fibrinogen	150~400 mg/dL	393
BUN	8~20 mg/dL	18.8
TG	35~150 mg/dL	268
HDL-C	40~100 mg/dL	47
LDL-C	70~140 mg/dL	124
CRP	<0.30 mg/dL	0.21
FT4	0.93~1.75 ng/dL	0.78
FT3	2.5~3.5 pg/mL	2.6
TSH	0.65~5.55 μU/mL	8.1
ApoA1	126~165 mg/dL	142
ApoA2	24.6~33.3 mg/dL	24
ApoB	66~101 mg/dL	96
ApoC2	1.5~3.8 mg/dL	3.4
ApoC3	5.4~9.0 mg/dL	10
ApoE	2.8~4.6 mg/dL	2.4

**Table 2:** Main changes induced by treatment NA; not available

Item	Start	3 months	6 months	normal range
Severity	0.67	0.54	0.39	<1.19
Extent	0.80%	0.13%	0.00%	<14.2%
Ratio	0.04	0.01	0	<2.22
Platelet	24.3	20.9	23.7	15~40×10^4/µL
D-dimer	1	N.A.	0.7	< 1.0 µg/mL
LA thrombi	2×18 mm	2×15 mm	0×0 mm	0×0 mm

## Discussion

This manuscript describes a case involving a thrombus in the RLPV that intruded into the left atrium. The left atrial thrombus was connected to the thrombus in a branch of the RLPV, which may have supported the position of the left atrial thrombus. This author published two case reports describing similar thrombi in patients with malignant lymphoma [18] and hypertension [17]. One prior case [18] could not evaluate the effects of edoxaban on the thrombus because the patient died of malignant lymphoma prior to receiving edoxaban treatment. However, the patient described herein did not suffer from severe disease. In the latter case [17], the author could not evaluate the effects of three months of rivaroxaban treatment on the thrombus but could only evaluate whether the thrombus was resolved by six months of treatment. Therefore, it was not possible to assess the speed of resolution of the left atrial thrombus. In the current case study, after three months of edoxaban treatment, TEE revealed shortening of the left atrial thrombus; after six months of edoxaban treatment, TEE revealed no thrombus in the left atrium. However, the connected thrombus in the RLPV remained present. Thus, we can conclude that this case involved a thrombus of the RLPV that intruded into the left atrium, with the left atrial thrombus gradually resolving after six months of edoxaban treatment.

Although six months of DOAC treatment was reported to ameliorate cerebral blood flow, as determined using brain perfusion SPECT, the effect of three months of DOAC treatment was uncertain. In the described patient, cerebral blood flow, as measured by brain perfusion SPECT, gradually improved from 0.67 for severity, 0.8% for extent, and 0.04 for ratio to 0.39 for severity, 0.0% for extent, and 0.00 for ratio after six months of edoxaban treatment, indicating that edoxaban can prevent or cure dementia or Alzheimer’s disease [19]. DOACs, as a whole, were reported not to prevent dementia in patients with AF [20]; however, the author reports that edoxaban had a special effect to ameliorate cerebral blood flow compared to rivaroxaban [15]. Further studies are needed to clarify these findings.

To conclude, the author reported that six months of treatment with DOACs palliated cerebral blood flow in patients with PVTs; then, it was noted that edoxaban has the potential to ameliorate cerebral blood flow [15]. DOACs might improve dementia through the recovery of cerebral blood flow, among which edoxaban may strongly improve both cerebral blood flow [15] and prevent dementia [19]. Additionally, most importantly, many of the author's patients had no AF, indicating the crucial possibility of preventing and improving dementia for patients without AF, i.e., for all older individuals. The present case study demonstrated that edoxaban improved cerebral blood flow in a patient without AF, indicating that edoxaban can prevent dementia in patients without AF. Apixaban and edoxaban have been shown to prevent dementia in patients with AF [19]. Moreover, apixaban and edoxaban may ameliorate dementia in dementia patients without AF by improving cerebral blood flow. More work is needed before edoxaban and apixaban can be utilized for dementia treatment in patients.

## Conclusions

Edoxaban gradually resolved a left atrial thrombus without the occurrence of ischemic stroke. Edoxaban ameliorated cerebral blood flow after six months of treatment, as measured via brain perfusion SPECT, in a patient without AF. Pulmonary vein and left atrial thrombi may decrease cerebral blood flow by releasing microclots, but can be ameliorated by edoxaban. To clarify these relationships, further studies are needed.
